# Supplementary material for: Reactive oxygen detoxification contributes to Mycobacterium abscessus antibiotic survival
Source: eLife. 2026 Jul 21;14:RP104944. doi: 10.7554/eLife.104944 (PMC13387744; doi:10.7554/eLife.104944)
Supplement: Peer review history

# Editor report: eLife Assessment

**DOI:** 10.7554/eLife.104944.4.sa0

**Authors:** Amit Singh

**Affiliations:** Indian Institute of Science India

**Keywords:** Solid, Valuable

Using a transposon sequencing (TN-seq) approach, the authors identified key genetic determinants of drug tolerance in Mycobacterium abscessus. Given that M. abscessus is inherently resistant to multiple antibiotics, this **valuable** study makes a significant contribution by uncovering how antibiotic tolerance is linked to reactive oxygen species (ROS) in this non-tuberculous mycobacterial (NTM) species. The **solid** findings further strengthen the growing evidence that ROS play a central role in the mechanism of antibiotic action and tolerance in mycobacteria. However, the use of words persistence or tolerance should follow the consensus definition given in the Balaban 2019 Nat Rev Micro paper.

# Referee report: Reviewer #2 (Public review)

**DOI:** 10.7554/eLife.104944.4.sa1

Summary:

The work set out to better understand the phenomenon of antibiotic persistence in mycobacteria. Three new observations are made using the pathogenic Mycobacterium abscessus as an experimental system: phenotypic tolerance involves suppression of ROS, protein synthesis inhibitors can be lethal for this bacterium, and levofloxacin lethality is unaffected by deletion of catalase, suggesting that this quinolone does not kill via ROS.

Strengths:

The ROS experiments are supported in three ways: measurement of ROS by a fluorescent probe, deletion of catalase increases lethality of selected antibiotics, and a hypoxia model suppresses antibiotic lethality. A variety of antibiotics are examined, and transposon mutagenesis identifies several genes involved in phenotypic tolerance, including one that encodes catalase. The methods are adequate for making these statements.

Overall impact:

Showing that ROS accumulation is suppressed during phenotypic tolerance, while expected, adds to the examples of the protective effects of low ROS levels. Moreover, the work, along with a few others, extends the idea of antibiotic involvement with ROS to mycobacteria. These observations help solidify the field. The work raises an important unanswered question: why are rifampicin and many protein synthesis inhibitors bacteriostatic with *E. coli* but bactericidal with pathogenic mycobacteria?

Comments on revisions:

I call attention to word choice, because it can indicate how familiar the authors are with the field. An issue that caught my attention was the use of the words persistence and tolerance, because they are not uniformly used in the generally accepted way (see Balaban 2019 Nat Rev Micro). In this consensus statement persistence refers specifically to a subpopulation and as such has survival kinetics that are distinct from those seen with tolerance, a phenomenon that refers to the entire population. I notice that the Balaban paper is not in the reference list. My suggestion is to take a look at the Balaban paper and then examine every use of the words tolerance and persistence in the manuscript to be sure that they fit the Balaban definition.

# Author comment: Author response

**DOI:** 10.7554/eLife.104944.4.sa2

**Authors:** Nicholas A Bates, Ronald Rodriguez, Rama Drwich, Abigail Ray, Sarah A Stanley, Bennett H Penn

**Affiliations:** University of California Davis Medical Center Davis United States; University of California, Berkeley Berkeley United States; University of California Davis Medical Center Davis United States; University of California Davis Medical Center Davis United States; University of California, Berkeley Berkeley United States; University of California Davis Medical Center Davis United States

The following is the authors’ response to the previous reviews

**Public Reviews:**

**Reviewer #1 (Public review):**

Summary:

Persistence is a phenomenon by which genetically susceptible cells are able to survive exposure to high concentrations of antibiotics. This is especially a major problem when treating infections caused by slow growing mycobacteria such as *M. tuberculosis* and *M. abscessus*. Studies on the mechanisms adopted by the persisting bacteria to survive and evade antibiotic killing can potentially lead to faster and more effective treatment strategies.

To address this, in this study, the authors have used a transposon mutagenesis based sequencing approach to identify the genetic determinants of antibiotic persistence in M. abscessus. To enrich for persisters they employed conditions, that have been reported previously to increase persister frequency - nutrient starvation, to facilitate genetic screening for this phenotype. M.abs transposon library was grown in nutrient rich or nutrient depleted conditions and exposed to TIG/LZD for 6 days, following which Tnseq was carried out to identify genes involved in spontaneous (nutrient rich) or starvationinduced conditions. About 60% of the persistence hits were required in both the conditions. Pathway analysis revealed enrichment for genes involved in detoxification of nitrosative, oxidative, DNA damage and proteostasis stress. The authors then decided to validate the findings by constructing deletions of 5 different targets (pafA, katG, recR, blaR, Mab_1456c) and tested the persistence phenotype of these strains. Rather surprisingly only 2 of the 5 hits (katG and pafA) exhibited a significant persistence defect when compared to wild type upon exposure to TIG/LZD and this was complemented using an integrative construct. The authors then investigated the specificity of delta-katG susceptibility against different antibiotic classes and demonstrated increased killing by rifabutin. The katG phenotype was shown to be mediated through the production of oxidative stress which was reverted when the bacterial cells were cultured under hypoxic conditions. Interestingly, when testing the role of katG in other clinical strains of Mab, the phenotype was observed only in one of the clinical strains demonstrating that there might be alternative anti-oxidative stress defense mechanisms operating in some clinical strains.

Strengths:

While the role of ROS in antibiotic mediated killing of mycobacterial cells have been studied to some extent, this paper presents some new findings with regards to genetic analysis of M. abscessus susceptibility, especially against clinically used antibiotics, which makes it useful. Also, the attempts to validate their observations in clinical isolates is appreciated.

Weaknesses:

Amongst the 5 shortlisted candidates from the screen, only 2 showed marginal phenotypes which limits the impact of the screening approach.

We appreciate the reviewer’s comments, but we note that 4 out of 5 genes displayed phenotypes concordant with findings of the Tn-Seq data, with *katG* and *pafA*, as well as *MAB_1456c* (during starvation only) and *blaR* (in rich media only) having decreased survival as shown in Figure 3A-D. We do agree that some of the phenotypes were more modest in a single-mutant context than in the pooled Tn-Seq screen. In addition, several mutants that had modest changes in survival also showed profound defects in resuming growth after removal of antibiotics, with the *pafA* mutants particularly impaired. (Figure 3 - figure supplement 1).

While the role of KatG mediated detoxification of ROS and involvement of ROS in antibiotic killing was well demonstrated, the lack of replication of this phenotype in some of the clinical isolates limits the significance of these findings.

While the role of *katG* varied among strains, the antibiotic-induced accumulation of ROS was seen in all three strains (Figure 6A). This suggests that in some strains other ROS-detoxification pathways are able to compensate for the loss of *katG*.

(Figure 2—figure supplements 1–3)

Figure 1—figure supplement 1.

**Reviewer #2 (Public review):**

Summary:

The work set out to better understand the phenomenon of antibiotic persistence in mycobacteria. Three new observations are made using the pathogenic Mycobacterium abscessus as an experimental system: phenotypic tolerance involves suppression of ROS, protein synthesis inhibitors can be lethal for this bacterium, and levofloxacin lethality is unaffected by deletion of catalase, suggesting that this quinolone does not kill via ROS.

Strengths:

The ROS experiments are supported in three ways: measurement of ROS by a fluorescent probe, deletion of catalase increases lethality of selected antibiotics, and a hypoxia model suppresses antibiotic lethality. A variety of antibiotics are examined, and transposon mutagenesis identifies several genes involved in phenotypic tolerance, including one that encodes catalase. The methods are adequate for making these statements.

Weaknesses:

The work can be improved by a more comprehensive treatment of prior work, especially comparison of *E. coli* work with mycobacterial studies.

Moreover, the work still has some technical issues to fix regarding description of the methods, supplementary material, and reference formating.

See detailed responses below.

Overall impact: Showing that ROS accumulation is suppressed during phenotypic tolerance, while expected, adds to the examples of the protective effects of low ROS levels. Moreover, the work, along with a few others, extends the idea of antibiotic involvement with ROS to mycobacteria. These are fieldsolidifying observations.

Comments on revisions:

The authors have moved this paper along nicely. I have a few general thoughts.

It would be helpful to have more references to specific figures and panels listed in the text to make reading easier.

Text modified to add more figure references.

(1) I would suggest adding a statement about the importance of the work. From my perspective, the work shows the general nature of many statements derived from work with *E. coli*. This is important. The abstract says this overall, but a final sentence in the abstract would make it clear to all readers.

We appreciate the suggestion and have added a line to the abstract.

(2) The paper describes properties that may be peculiar to mycobacteria. If the authors agree, I would suggest some stress on the differences from *E. coli*. Also, I would place more stress on novel findings. This might be done in a section called Concluding Remarks. The paper by Shee 2022 AAC could be helpful in phrasing general properties.

We have added mention of this in the discussion (lines 354-356).

(3) Several aspects still need work to be of publication quality. Examples are the materials table and the presentation of supplementary material. Reference formatting also needs attention.

We respond to the specific details below.

**Reviewer #3 (Public review):**

Summary:

The manuscript demonstrates that starvation induces persister formation in M. abscesses.

They also utilized Tn-Seq for the identification of genes involved in persistence. They identified the role of catalase-peroxidase KatG in preventing death from translation inhibitors Tigecycline and Linezolid. They further demonstrated that a combination of these translation inhibitors leads to the generation of ROS in PBS-starved cells.

Strengths:

The authors used high-throughput genomics-based methods for identification of genes playing a role in persistence.

Weaknesses:

The findings could not be validated in clinical strains.

Comments on revisions: No more comments for the authors.

**Recommendations for the authors:**

**Reviewer #1 (Recommendations for the authors):**

The authors are strongly encouraged to check the references. There is some systematic error in the citations of references. Started to list but then they were too many.

For example Ln 51, Ref #11 cited, should be #10. Ln 59, #18 is wrongly cited. Should be - Ln 104. Ref #27 wrongly cited.

Ref #26 and #28 identical.

Even in discussion section a lot of references are mis-cited.

We very much appreciate the reviewer catching this issue with the import of our references and we have corrected this.

**Reviewer #2 (Recommendations for the authors):**

Below I have listed comments on specific issues that I hope are useful during revision.

Line 21 population is singular

Text modified

Line 21 comma after antibiotic (subordinate clause) Line

Text modified

25 is how singular?

Text modified

Impression of abstract: the work seems to confirm and therefore generalize concepts derived from studies with *E. coli*. If the authors agree, such a statement would be appropriate as a final sentence. I would also look for novel features to stress in the abstract.

Line 41 this challenge is vague

Text modified

Line 43 comma such as (also comma at the end of the parenthetical statement). This type of comma error is common throughout the manuscript and slows reading.

Text modified

Line 60 paradoxically. Is this the best concept? Or is it the natural effect of evolution (assuming that mycobacteria or their ancestors were exposed to environmental antibiotics)?

It is certainly problematic for clearing infection.

Text not modified.

Line 63 highlighted uncertainties ... meaning is unclear especially since you may have changed what "model" is referring to.

Text modified

Line 66 models.... Do you really mean systems? Models of what?

This refers to mechanistic models. Text not modified.

Line 67 arrest cell division. This is written as if it were true. Does the evidence point specifically to cell division or perhaps more accurately suppression of metabolism (see Ye et al 2025 mBio).

Both have been postulated as important. Text modified to add concept of metabolism

... targeted by antibiotics non-essential... Do you think that antibiotics work by inactivating essential targets? That seems overly simplistic, as lethal action is more likely the metabolic response to the damage caused. By the end of the paragraph you come around to this view, but you have already misdirected the reader. The reader is not sure what to believe. Line 70 note that there are many inhibitors of transcription and translation that only block growth, they do not rapidly kill cells

There can be both direct, and indirect secondary killing mechanisms. We devote a significant portion of the Discussion section to this topic.

Line 71 debate. There was indeed a debate, but reference 22 is not a valid citation for this. I think you mislead the reader by not accurately describing the debate. It was basically about the inability of Kim Lewis and James Imlay to reproduce the work of ref. 22. A great deal of prior work and then subsequent work showed that the challenge to ref. 22 lacked substance.

(1) Text modified to fix an error in the citation number related to direct β-lactam-mediated lysis.

(2) We agree that there is a great deal of data supporting antibiotic-induced ROS as important for bactericidal activity in many circumstances and do not argue otherwise. This sentence points out that over the years the paradigm for how antibiotics kill bacteria has evolved.

Line 80. It seems you are starting a new topic here. What about beginning a new paragraph?

The paragraph introduces mycobacteria of which *Mabs* is one. Text not modified.

Line 85 delete the comma: it implies a compound sentence that is not delivered.

Text modified.

Line 109 screen singular

Text modified.

Line 156 these conditions is imprecise and vague

Conditions were described in paragraph above in the manuscript. Text not modified.

Fig 2 it would be helpful to more clearly define the meaning of the coordinates

Text modified.

Line 230 and throughout please indicate the location of the data being cited for rapid reader reference

Text modified.

Lines 315-323 You could use this paragraph as the first of the Discussion. Some readers prefer to read the Discussion before the results. For them, a summary at the beginning of the Discussion is useful.

Text modified.

Line 328 without underlying mechanism... for *E. coli* refer to Zeng PNAS 2022. Depending on when the final version of this paper happens, there should be a figure in a Zhao Zhu mLife paper on purA that will have been published. Since it is not yet available, it cannot be cited.

We agree that the Zeng et al study is interesting and have added this reference to our discussion. However, these findings related to broad Crp-regulated tolerance actually underscore the point that we are making: that there are multiple factors (Crp, RelA, Lon, TisB, MazE, others) that mediate antibiotic tolerance.

Line 339 where are the data?

These data are in Figure 5, panels C, D. We have clarified the text to indicate that only a single agent from each of these classes was tested.

Line 346 here you are summarizing evidence for ROS in killing mycobacteria. You should include the moxifloxacin study by Shee et al 2022 AAC.

Reference added.

Line 348 refer to James Collins' work with *E. coli* in which his lab examined agents with a variety of mechanisms. There seems to be a fundamental difference between *E. coli* and mycobacteria with respect to rifampicin, a strictly static agent in *E. coli* but clearly lethal in mycobacteria. Note that chloramphenicol is static in *E. coli* and blocks ROS production. What does it do in mycobacteria? A brief discussion of this difference might be relevant at line 362

Text modified.

Lines 364-368 Here the idea might be simply that there are two modes of killing, one that is a direct extension of class-specific damage (chromosome fragmentation with fluoroquinolones, for example, or cell lysis by beta-lactams) and a second that is a metabolic response to the antibiotic damage (ROS accumulation). The second type is not class specific. Within this context, the mycobacterial killing by rifampicin might be a class-specific extension of inhibition of transcription that does not occur in *E. coli*.

Agreed, text modified to include this.

Line 400 The Key Resource table is not of publication quality. Precision and repeatability can be improved by spelling out the name of the vendor and its location (City, Country). In the present case, use of BD is lab jargon.

We appreciate the reviewer’s precision. However, this is actually not lab jargon. Becton, Dickinson and Company now refers to itself as BD (see https://www.bd.com/en-us), and the American Type Culture Collection now refers to itself as ATCC (see https://www.atcc.org/about-us/who-we-are).

Line 639 It would be good to have experienced colleagues critically review the manuscript, especially for English usage. Listing those persons here adds to the credibility of the work

Text not changed.

References: please refer to the journal style. Here you use italic for titles and scientific names, thereby obscuring the scientific names. Normally article titles are not italic and scientific names are ALWAYS italic unless prohibited by journal style.

Our reference format is concordant with eLife submission guidelines, and all references are reformatted by the journal at the time of final publication (see https://elifesciences.org/insideelife/a43f95ca/elife-references-yes-we-take-any-format-no-we-re-not-rekeying).

Supplemental Material: Please refer to journal style. Normally this is a stand-alone document that includes a title page and carefully crafted figure legends. Supplemental figures would be numbered as 1, 2, ... A professional appearing Supplemental Material section shows author publication experience not obvious in other parts of the paper. The text indicated MIC determinations. I would like to see a table of MIC values.

(1) MIC table added as Supplemental Table 5.

(2) The Supplemental figures are submitted and named in accordance with eLife instructions. Please note that for eLife, there is not a stand-alone supplementary figure section with a title page as you are requesting, but instead the figure supplements for each figure are provided as online files linked to each figure.
